# Wild snakes harbor West Nile virus

**DOI:** 10.1016/j.onehlt.2016.09.003

**Published:** 2016-09-22

**Authors:** C.R. Dahlin, D.F. Hughes, W.E. Meshaka, C. Coleman, J.D. Henning

**Affiliations:** a Department of Biology, University of Pittsburgh at Johnstown, Johnstown, PA 15904, United States; b Department of Biological Sciences, University of Texas at El Paso, 500 West University Avenue, El Paso, TX 79968, United States; c Section of Zoology and Botany, State Museum of Pennsylvania, 300 North Street, Harrisburg, PA 17120, United States

**Keywords:** West Nile virus, Snake, RNA, Flavirus, Reptile, Virus

## Abstract

West Nile virus (WNV) has a complex eco-epidemiology with birds acting as reservoirs and hosts for the virus. Less well understood is the role of reptiles, especially in wild populations. The goal of our study was to determine whether a wild population of snakes in Pennsylvania harbored WNV. Six species of snakes were orally sampled in the summer of 2013 and were tested for the presence of WNV viral RNA using RT-PCR. Two Eastern Garter Snakes, *Thamnophis sirtalis sirtalis* tested positive for viral RNA (2/123, 1.62%). These results indicate a possible role for snakes in the complex transmission cycle of WNV.

West Nile virus (WNV) has posed enormous health problems both to the public and wildlife since it arrived in the United States in 1999. Costs to wildlife have been arguably higher as compared to humans; millions of birds have died from WNV and for some species and locales > 50% of the population has perished [1]. Although the importance of birds in WNV transmission is well-understood, the eco-epidemiology of WNV is complex and the role that other vertebrates might play in the transmission cycle is virtually unknown. It has been shown that reptiles play a role in the transmission cycle of other flaviviruses, such as western and eastern equine encephalitis virus and St. Louis encephalitis virus [2], [3], [4], [5], [6], [7], [8], [9], [10]. In laboratory settings, several species including Green Iguanas (*Iguana iguana*), Eastern Garter Snakes, Red-Ear Sliders (*Trachemys scripta elegans*), North American Bullfrogs (*Lithobates catesbeianus*) and Western Fence-Lizards (*Sceloporus occidentalis*) develop detectable viremia titers of WNV [11], [12], [13], and snakes have died after developing high titers of WNV [12]. Among the herpetofauna, WNV has been shown to be most pathogenic in Crocodilians, causing high titer viremias in several countries (For a review see [14]. Despite the mounting evidence that reptiles may indeed play a role in WNV ecology, knowledge of WNV in reptiles generally comes from experimental infections or farmed individuals rather than wild populations. Nevertheless, a recent study found that WNV antibodies in wild Morelet's crocodiles (*Crocodylus moreletii*) in Mexico were as high as 41%, indicating that studies on wild animals can provide novel insights into the eco-epidemiology of WNV [15].

In order to better understand the eco-epidemiology of WNV in non-avian wildlife, we set out to assess the presence of WNV RNA in wild snakes. We conducted our study in Pennsylvania, a state that has harbored WNV since 2000 and exhibits fluctuating levels of WNV [16]. For example, 2003 represented a high year with all 67 counties testing positive for WNV, while 2013, the year of our study, was intermediate with 42 positive counties and 5.9% of mosquito pools testing positive. West Nile virus increased again in 2015 with 56 positive counties and 14.5% of mosquito pools testing positive [16]. The presence of WNV in Pennsylvania's wildlife has been only monitored through sporadic testing of live birds [17] and to our knowledge there are no reports on WNV testing in Pennsylvania reptiles.

Snakes were sampled between May and July 2013 at one wetland site and various grassland sites at Powdermill Nature Reserve (PNR), which is a field station owned and operated by the Carnegie Museum of Natural History (40°10′N, 79°16′W; elevation 400 m). The reserve is 856.2 ha in size and located in Rector, Westmoreland County, Pennsylvania. Snake populations have been monitored at PNR since 2002 using 1 × 3 m corrugated metal cover boards [18]. In the 2013 sampling season snakes were being monitored for ecological purposes under Permit No. 119 of the Pennsylvania Fish and Boat Commission, which also allowed us to opportunistically sample snakes for WNV. All captured snakes have been fitted with AVID Passive Integrated Transponder Tags [18], which allowed for individual identification of previously captured and new snakes. Captured snakes were individually marked, species and sex identified, and orally swabbed with a long sterile cotton applicator (BD CultureSwab + Transportation System). The swabs were stored with Tris Borate Ethylenediaminetetraacetic Acid buffer and maintained at − 20 °C to preserve viral RNA.

Viral RNA was extracted and PCR analysis was done as previously described [17]. Briefly, viral RNA was extracted from oral samples by using the QIAamp viral RNA kit (QIAGEN, Valencia, Calif.) [19], [20]. RNA was eluted from the QIAgen columns in a final volume of 100 μL of elution buffer and was stored at − 80 °C until used. The PCR primers of the NS5 gene were used to detect WNV [21]. The NS5 gene is shared among the Japanese encephalitis virus complex, and thus we cannot say with 100% certainty that our positive detections of flavivirus RNA was indicative of WNV as opposed to a related virus. WNV is generally more common in PA and thus more likely to be detected using this methodology. Viral RNA was converted to cDNA by utilizing the PowRSybr One-Step RNA to CT Kit from Life Technologies. Five microliters of RNA and 50 pmol of each primer were used in a 50-μL total reaction volume by following the manufacturer's protocol with cycling times as indicated by Lanciotti et al. [21]. After the RT-PCR was performed, a 5-μL portion was analyzed by agarose gel electrophoresis and the DNA was visualized by ethidium bromide staining. WNV RNA, strain NY99, obtained from the reference collection maintained at the Division of Vector-Borne Infectious Diseases, Centers for Disease Control and Prevention (CDC) was used as a positive control in the RT-PCR assay.

To assess the confidence of no infection in the absence of disease, we calculated adjusted Wald intervals and LaPlace point estimates for all species with samples sizes that were > 10 individuals [22].

Oral samples consisted of 123 individuals from six species of snakes (Northern Ringneck Snake, *Diadophis punctatus punctatus*, Northern Water Snake, *Nerodia sipedon sipedon*, Midland Rat Snake, *Scotophis spiloides*, Northern Brown Snake, *Storeria dekayi dekayi*, Northern Redbelly Snake, *Storeria occipitomaculata occipitomaculata*, and Eastern Garter Snake) (Table 1). The Eastern Garter Snake was the most commonly encountered species during our sampling, representing 59% of the snakes, and the source of the two positive samples (Table 1). Confidence intervals (CE) for the two species with > 10 sampled individuals, which was the Eastern Garter Snake and the Northern Redbelly Snake, had a low CE that ranged between 0 and 0.002 and a high CE that ranged between 0.09 and 0.1 (Table 2). Point estimates ranged from 0.03 to 0.04 (Table 2).

**Table 1:** Sampling information and WNV positivity for snake species sampled at the Powdermill Nature Reserve, Westmoreland County, Pennsylvania, USA, in 2013.

Species	Sample size	Sex of snake			
		Male	Female	Unknown	WNV positive
*Diadophis p. punctatus*	7	5	2	0	0
*Nerodia s. sipedon*	6	3	3	0	0
*Scotophis spiloides*	4	2	1	1	0
*Storeria d. dekayi*	2	0	2	0	0
*Storeria o. occipitomaculata*	30	1	29	0	0
*Thamnophis s. sirtalis*	73	18	54	1	2
Unknown	1	1	0	0	0
Total	123	30	91	2	2

**Table 2:** Confidence intervals and point estimates for snake species with samples > 10 from the Powdermill Nature Reserve, Westmoreland County, Pennsylvania, USA, in 2013.

Species	N	N WNV positive	Low CE	High CE	Margin of Error	Point Est.
*Storeria s. occipitomaculata*	30	0	0	0.09	0.06	0.03
*Thamnophis s. sirtalis*	73	2	0.002	0.1	0.05	0.04

Our study demonstrated that WNV is present in wild snakes and WNV RNA can be successfully detected from oral swabs. Although only two individuals tested positive (0.016%), these samples were collected from an area now known to have relatively low rates of infection in birds (0.009%) [17]. Thus, while the confidence intervals and point estimates indicated that overall levels of WNV RNA in snakes were low at PNR, the current rates of positivity are comparable to avian rates. In addition, while Westmoreland was one of the 42 counties in which WNV was detected in 2013, there were no human or avian infections found and only 1.6% of the mosquitos in Westmoreland County tested positive, as opposed to 9% statewide [16]. Since rates of WNV are so low in Westmoreland County, it raises the possibility that the RNA we found is evidence of persistence, for which evidence has been found in other arboviruses [14]. Our study also contrasts with a study of Eastern Massasaugas, *Sistrurus catenatus catenatus*, in which none of the 21 sampled individuals tested positive for WNV [23].

A question that remains unaddressed from our study is; what is the role of snakes in the eco-epidemiology of WNV? Some bird species reach sufficient viremia titres (10^4^–10^5^ PFU/mL) of infectious virus, thus acting as “competent amplifying hosts” [24], [25]. Although mammals and reptiles can act as hosts, they are generally much less competent hosts than many bird species [26]. Our study was unable to assess the oral samples for infectious virus, and therefore could not determine viremia titer. However, experimental data on Eastern Garter Snakes has indicated this species can reach detectable viremia titers once infected (5/9 individuals became viremic with up to 10^5^ PFU/mL serum), and that some individuals can sustain viremia for up to 11 days [12]. These findings suggest that Eastern Garter Snakes may not only harbor WNV, but may also serve as competent hosts for the *Culex* mosquitos that live in the East [26]. An 11 day period of viremia [12] is longer than the 1–4 day periods that have been observed in other vertebrates [11], [27], [28], [29], which may increase the potential for transmission from infected snakes, highlighting the need for further research [26]. In addition, Steinman et al. (2006) found that some snakes exhibited signs of illness prior to death such as weakness and immobility, which could increase their likelihood of being predated upon in the wild. A variety of raptors and mammals will readily prey upon Eastern Garter Snakes [30]. Direct, non-vector transmission has been found to be ecologically important in several species [26], [31], [32], and it will be worthwhile to investigate whether reptiles have the potential to orally infect predatory birds and mammals in the wild if eaten.

Both positive samples in our study originated in Eastern Garter Snakes, which are the most dominant species in the grasslands at PNR [33]. Currently we have not sampled a sufficient number of snakes across species to determine whether this species harbors more WNV, or if the larger rates at which the Eastern Garter Snake were encountered led to the increased probability of WNV being detected. It is also important to note that the six species that we tested represent only a fraction of herpetofauna across the USA. Thus, although our work represents an important step in WNV research in wild reptiles, the inclusion of more snake species at sites with higher rates of WNV, as well as other reptiles would greatly improve our understanding of the role of other non-avian vertebrates in WNV transmission. Another important next step will be to determine whether snakes in the wild have active viremia, which would clarify the contribution of snakes to WNV transmission and improve our understanding of the eco-epidemiology of WNV, both of which have implications for human health.

## Conflict of interest statement

Conflicts of interest: none.
